# Inflammation-Related Mechanisms in Chronic Kidney Disease Prediction, Progression, and Outcome

**DOI:** 10.1155/2018/2180373

**Published:** 2018-09-06

**Authors:** Simona Mihai, Elena Codrici, Ionela Daniela Popescu, Ana-Maria Enciu, Lucian Albulescu, Laura Georgiana Necula, Cristina Mambet, Gabriela Anton, Cristiana Tanase

**Affiliations:** ^1^Victor Babes National Institute of Pathology, 050096 Bucharest, Romania; ^2^Carol Davila University of Medicine and Pharmacy, 050474 Bucharest, Romania; ^3^Stefan S. Nicolau Institute of Virology, Molecular Virology Department, 030304 Bucharest, Romania; ^4^Titu Maiorescu University, Faculty of Medicine, 040441 Bucharest, Romania

## Abstract

Persistent, low-grade inflammation is now considered a hallmark feature of chronic kidney disease (CKD), being involved in the development of all-cause mortality of these patients. Although substantial improvements have been made in clinical care, CKD remains a major public health burden, affecting 10–15% of the population, and its prevalence is constantly growing. Due to its insidious nature, CKD is rarely diagnosed in early stages, and once developed, its progression is unfortunately irreversible. There are many factors that contribute to the setting of the inflammatory status in CKD, including increased production of proinflammatory cytokines, oxidative stress and acidosis, chronic and recurrent infections, altered metabolism of adipose tissue, and last but not least, gut microbiota dysbiosis, an underestimated source of microinflammation. In this scenario, a huge step forward was made by the increasing progression of omics approaches, specially designed for identification of biomarkers useful for early diagnostic and follow-up. Recent omics advances could provide novel insights in deciphering the disease pathophysiology; thus, identification of circulating biomarker panels using state-of-the-art proteomic technologies could improve CKD early diagnosis, monitoring, and prognostics. This review aims to summarize the recent knowledge regarding the relationship between inflammation and CKD, highlighting the current proteomic approaches, as well as the inflammasomes and gut microbiota dysbiosis involvement in the setting of CKD, culminating with the troubling bidirectional connection between CKD and renal malignancy, raised on the background of an inflammatory condition.

## 1. Introduction

Low-grade chronic systemic inflammation is a condition characterized by persistent, low to moderate levels of circulating inflammation markers. It has been long associated with coronary heart disease [1], metabolic syndrome, diabetes [2], and aging [3]. However, not only elderly pathologies are associated with the presence of low systemic inflammation. As systemic inflammation has also been reported in children and teenagers with weight problems [4], it is now clear that the persistence of the underlying condition and molecular mechanisms that trigger it should be taken into consideration in tandem with low chronic inflammation.

Whether inflammation is either a trigger or a result of a chronic underlying condition is an intensely studied topic. Studies on the impact of chronic inflammation on early stages of disease development, as well as the impact of early life nutrition on the adult inflammatory status, greatly extended the knowledge in the field (reviewed in [5]). Emergence of inflammation in childhood has been associated with obesity [6], diet [7], enteral infections [8], and even social stress [9]. Gene polymorphisms of inflammatory markers [4, 10] and/or inflammasome components [11] are also determinants of the inflammatory response of patients in the face of chronic injuries.

The main sources of inflammatory cytokines are circulating monocytes and endothelial cells. Ubiquitous distribution of the latter could be responsible for the wide-spread impact of inflammation in almost all organs, including the bone. The kidney receives 25% of the entire blood volume, without having the benefit of antioxidant, detoxifying, and anti-inflammatory defence mechanisms developed by other intensely vascularized tissues, such as hepatic tissue. Hence, the kidney stands as a vulnerable target in front of persistent aggression.

Chronic kidney disease (CKD) is defined as “abnormalities of the kidney structure or function, present for more than 3 months, with implications for health” [12]. There is no question that inflammation plays a part in CKD progression and outcome [13], but the link between initiation of the disease and inflammation is still under debate. Similar to other chronic diseases, CKD is accompanied by a low-grade chronic inflammation, to which the kidney is vulnerable in more than one way, as discussed in the following section. Notably, distant sources of inflammation, such as a dysregulation of gut microbiota [14] or alteration of intestinal barrier [15], can negatively impact on progression of CKD and uremia-associated complications. Relationship between diet, gut microbiota, and CKD will be further detailed in one of the sections of this review. A particular issue to be addressed in the present review is the relationship between CKD, chronic inflammation, and malignancy. Similar to other chronic diseases, various types of cancer (colorectal [16], pancreatic [17], breast [18], aggressive prostate [19], lung [20], ovarian [21], or brain [22]) are associated with underlying chronic inflammation. Systemic inflammation has also been associated with renal cancers, especially in terms of prognosis [23–25], being as well a promoter of cell transformation and metastasis [26]. This review will look into more detail whether progression of CDK towards malignancy is a possibility that a clinician should consider in the context of systemic inflammation. Finally, the review will conclude with updates regarding proteomic studies of biomarkers for diagnostic, for accurate stratification, or progression from one stage to another, discussed in the framework of global search for ideal biomarkers.

## 2. Vulnerability of Kidneys Facing Inflammation

The role of inflammation in CKD pathogenesis and progression has been recognized since the late 1990s, when the first provocative theory was launched, in which inflammation, via monocyte release of interleukin-1 (IL-1), the master cytokine of inflammation, was the starting point concerning the major complications and the increasing rate of mortality in patients undergoing chronic dialysis [27]. It has also been described how polymorphisms in the IL-1 gene cluster influence levels of IL-1 gene products, which were later encountered in various inflammatory disease states. Since then, there has been an exponential growth of interest in deciphering the role played by the inflammatory cytokines released in the uremic milieu of CKD, as independent predictors of morbidity and mortality in CKD patients. While the release of proinflammatory cytokines could determine favourable effects, persistent inflammation is recognized to promote adverse consequences.

There are many factors that contribute to chronic inflammatory status in CKD, including increased production of proinflammatory cytokines, oxidative stress and acidosis, chronic and recurrent infections, altered metabolism of adipose tissue, and intestinal dysbiosis.

Inflammatory activation in CKD seems to be also influenced by genetic and epigenetic conditions. Therefore, several approaches have been proposed to target inflammation in CKD, including lifestyle changes, drugs, and dialysis optimization [28].

The evidence obtained so far sustains that inflammation and inflammatory reactions of any cause can modify or interfere with the intrarenal microcirculatory regulation and perfusion distribution and can induce renal damage, thus enhancing CKD progression.

It is well recognized the uniqueness of microcirculation networks in kidneys, being essential to sustain the corticomedullary osmotic gradient for fluid absorption and urine concentration. Under physiological conditions, the distribution of intrarenal vasculature is heterogeneous, and the medulla resides in a hypoxic milieu; therefore, the energy deprivation is eluded by an avalanche of regulators, such as hormones and other vasoactive molecules (prostaglandins, endothelins, kinins, medullipin, nitric oxide, and other molecules), mostly synthesized in the medulla [29]. Regardless of the highly regulated microcirculatory balance that keeps the kidneys efficient, it has to be mentioned that any slight imbalance in the interaction amongst these molecules could alter kidney function, thus rendering kidneys vulnerable to the microenvironment.

Systemic or intrarenal inflammation contributes to deregulation of the microvascular response to its regulators and sustains the production of an array of tubular toxins, including reactive oxygen species (ROS), leading to tubular injury, nephron dropout, and the onset of CKD. Circulating proinflammatory cytokines activate intrarenal microvessels, particularly endothelial cells and leukocytes, resulting in a local amplification of proinflammatory factors and ROS. These processes affect cell-surface adhesion molecules and disrupt the glycocalyx layer. Endothelial barrier function, activation of coagulation system, and receptor-mediated vasoreactivity are also compromised. These inflammation-mediated alterations can induce irreversible tubular injury and nephron failure [30].

Oxidative stress and inflammation are inseparably linked, being major characteristics of CKD and drivers of CKD progression. Systemic inflammation presence and severity contributes to CKD-associated oxidative stress, which represents a condition in which generation of ROS surpasses the capacity of the antioxidant defence system [31].

The inflammatory microenvironment, mediated by cytokines, induces overexpression of reactive oxygen/nitrogen species, bioactive lipids, and adhesion molecules. Cytokines are also responsible for the promotion of aberrant matrix metabolism, proliferation of resident cells, and procoagulant activity of endothelium in the kidney. Cytokines control the inflammatory response and mediate some of their downstream effects through positive acute-phase proteins, such as C-reactive protein, fibrinogen, and albumin. In a recent study that analyses the association between a set of inflammatory biomarkers and progression of CKD in the Chronic Renal Insufficiency Cohort, the authors reported that elevated circulating levels of fibrinogen and TNF-*α* and decreased serum albumin are linked with the rapid loss of kidney function in patients with CKD, and these markers are independent predictors of CKD progression [32].

Systemic inflammation in end-stage renal disease is a well-recognized risk factor for the increased mortality in these patients and a catalyst for other complications, which are related to a premature aging phenotype, including muscle wasting, vascular calcification, and other forms of premature vascular disease, depression, osteoporosis, and frailty. Uremic inflammation is also involved in the aging process, such as telomere shortening, mitochondrial dysfunction, and altered nutrient sensing, which can have a direct effect on cellular and tissue function [33]. An in vitro study showed that circulating inflammatory monocytes from advanced CKD or hemodialysis patients transdifferentiate into osteoclasts and play a relevant role in mineral bone disorders. CKD patients, characterized by reduced renal function, frequently present an increased inflammatory state and skeletal abnormality [34].

Patients with CKD often display chronic increase in markers of inflammation, a condition that seems to be intensified by the disease progression and onset of hemodialysis. Systemic inflammation is related to malnutrition and muscle protein wasting and is involved in many morbidities including cardiovascular disease, the most common cause of mortality in this population. Investigation in the general population and other chronic disease cohorts demonstrated that an increase in habitual activity levels over a prolonged period may normalize the systemic inflammation. Furthermore, those populations with the highest baseline levels of systemic inflammation appear to have the greatest improvements from training [35]. Systemic inflammation, alongside with the loss of kidney function, can damage the resistance of the body to external and internal stressors, by reducing functional and structural tissue reserves and by impairing normal organ crosstalk, thus providing an explanation for the greatly increased risk of homeostatic breakdown in this population [35].

Overall, CKD patients show elevations in markers of chronic inflammation. Since inflammation, malnutrition, and protein-energy wasting are important contributors to mortality in CKD patients, any treatments which may positively influence these conditions should be taken into consideration [35].

Despite recent advances in the management of chronic kidney disease (CKD) and end-stage renal disease (ESRD), morbidity and mortality continue to be remarkably high in these patients. Persistent, low-grade inflammation has been recognized as an important component of the CKD scenario, leading to fibrosis and loss of renal function, and is playing a crucial role in the pathophysiology and progression of the disease, with a major impact on its complications [28].

## 3. Inflammasomes, Inflammation, and CKD

The inflammasomes have recently become the subject of intensive research, since they seem to play a major role in the pathogenic mechanisms in renal diseases. The inflammasomes are large, multiprotein complexes that could be induced by lipopolysaccharide (LPS). They were initially mentioned in 2002 as innate immune signaling pathways triggering activation of proinflammatory cytokines in response to various stimuli [36]. Innate immunity is an evolutionarily conserved system, the first line of host defence that supports homeostasis by regulating endogenous processes like inflammation and apoptosis. It relies on pattern recognition receptors (PRRs) that recognize damage-associated molecular patterns (DAMPs) and pathogen-associated molecular patterns (PAMPs) released in response to stress, tissue injury, or apoptosis [37]. Currently, several different classes of PRR families have been identified, which include transmembrane Toll-like receptors (TLRs), C-type lectin receptors (CLRs), retinoic acid-inducible gene (RIGs) receptors, intracellular Nod-like receptors (NLRs), and more recently included HIN-200 receptors. Extracellular PAMPs and DAMPs are recognized by TLRs and CLRs, while intracellular PAMPs are recognized by NLRs and RIGs [38, 39].

The activated innate immune system leads to activation of the prototypical proinflammatory signaling pathway, the best characterized being NF-κB (nuclear factor-kappa B) and AP-1 (activator protein-1), mainly based on the stimulation of multiple mediators, including proinflammatory cytokines such as interleukin-1 (IL-1) and tumour necrosis factor *α* (TNF-*α*). A decisive instrument in initiating the posttranscriptional processing and release of mature cytokines is represented by the development of the inflammasome complex. The human genome encodes 23 NLR proteins, from which the NLR with caspase recruitment domain (NLRC) are responsible of organizing an inflammasome complex and releasing of proinflammatory cytokines IL-1*β* and IL-18. There have been seven established NLRs that form an inflammasome complex: NLRP1 (NALP1), NLRP3 (NALP3 or cryopyrin), NLRP6, NLRP12, NLRC4 (with caspase recruitment domain or IPAF), AIM2 (absent in melanoma-2), and RIG-1 (retinoic acid inducible gene-1); however, the NLRP3 inflammasome is the best characterized in relation with renal diseases [40].

Activation of NLRP3 inflammasome is promoted by TLR activation, thereby triggering the NF-κB pathway and the proinflammatory cytokines being released as pro-IL-1*β* and pro-IL-18. In order to be converted into their active forms and be secreted, the cytokines require subsequent caspase cleavage, which determine NLRP3 to oligomerize in the presence of an adaptor molecule—ASC (apoptosis-associated speck-like protein containing a C-terminal caspase recruitment domain), and finally resulting in secretion of proinflammatory cytokines.

Despite the fact that recognition of a single unifying mechanism for the NLRP3 inflammasome activation remains elusive, several stimuli have been proposed that trigger assembly of the NLRP3 inflammasome, involving P2X_7_ (a ligand-gated ion channel) receptor, activated via ATP, with K^+^ efflux and reduction in intracellular K^+^; ROS production, the release of mitochondrial DNA and cardiolipin [41]. The role of ROS as essential secondary messengers signaling NLRP3 inflammasome activation was suggested in several studies, and various pathways have been anticipated to mediate ROS production by NLRP3 activators. It was speculated that K^+^ efflux could trigger ROS generation or other NLRP3 activators, such as uric acid crystals, alum, asbestos, and silica. Therefore, the so-called frustrated phagocytosis could be generated, being connected to ROS production, as well [42]. Various pathways have been proposed to mediate ROS production by NLRP3/NALP3 activators; however, the general picture of how NLRP3/NALP3 activators trigger ROS is still unclear.

Recent studies highlighted a broad role for inflammasome activation in renal diseases. Most of the studies regarding the role of NLRP3 have been performed on acute kidney injury (AKI) models, and fewer were done using models of CKD, due to the deficit of rodent models that could mimic the human CKD [43]. Among the various animal models, the unilateral ureteral obstruction (UUO) represents a suitable model of renal fibrosis, which was established as a model of CKD [44]. In a study using a UUO model, Vilaysane et al. concluded that inflammasome-dependent cytokines IL-1*β* and IL-18 were upregulated in association with caspase-1 activation; compared with wild-type mice, NLRP3^−/−^ mice expressed less tubular injury, inflammation, and fibrosis after UUO, which highlighted the activation of NLRP3 inflammasome [45]. Using the same UUO model, Pulskens et al. concluded that the absence of NLRP3 resulted in enhanced vascular leakage and interstitial edema and revealed no effect on fibrosis and inflammation. These data showed a noncanonical effect of NLRP3 inflammasome in protecting kidney integrity following progressive renal injury [46]. It is important to note that the UUO mice model does not represent an objective readout, and the significance of inflammasome in relation to CKD remains under critical debate. Several studies in mice models and still restricted studies in humans propose an extensive role for inflammasome activation in CKD. Surprisingly, individual components of the inflammasome activation could bring their own contribution to progressive renal injury [47].

In addition to their role in mediating acute kidney disease, the IL-1*β*/IL-18 axis could also be involved in the development of CKD itself and its related complications—accelerated vascular calcification, fibrosis, and sepsis. It was shown that vascular inflammation is related to vascular calcification, and the proinflammatory cytokine IL-18 was the most extensively studied component of the NLRP3 inflammasome in relation to CKD. The pathophysiology behind the elevated levels of IL-18 in CKD may be related to the levels of MCP-1 (monocyte chemoattractant protein-1), since eGFR was independently associated with the serum levels of MCP-1, thereby partially explaining the increased risk of cardiovascular complications in CKD [48].

Inflammation-related vascular injury and atherosclerotic plaques in CKD were also the subject of intense research, in relation to inflammasome cytokine-mediated NLRP3, while IL-18 levels were found to be correlated with aortic pulse wave velocity. The NLRP3 inflammasome is gaining recognition for its key role in the pathogenesis of CKD and its complications; however, understanding the different pathways through which the inflammasome contributes to their genesis will supply additional insights in providing potential therapeutic targets [40].

The current understanding of CKD is based on a broad range of studies, and the inflammasomes exert a major role as guardians of the body; nevertheless, their role in regulating the intestinal microbiota and the progression of major diseases has been recently depicted.

## 4. An Underestimated Source of Smouldering Inflammation—Gut Microbiota

Microbiota, the microbial community which colonizes the large intestine, is nowadays considered a symbiotic “supplementary organ,” consisting of trillions of microbes, which altogether contain several hundredfold more genes than the human genome. Microbiota, in terms of composition and metabolic activity, codevelops with the host even from birth and is subject to a complex interaction depending on host genome, diet, and lifestyle factors [49]. It was noticed that gut microbiota have fundamental roles in human health and disease, and the diversity of microbiota evolves over a person's life, shifting throughout childhood and adult life, continuing with elderly where it is poor in some taxonomic species, including Gram-negative Bacteroides species, and rich in Gram-positive Firmicutes species. Advances in sequencing technology (NGS) and bioinformatics have unravelled the complexity and diversity of human microbiome. Thus, the Human Microbiome Project has been launched in 2007 by the National Institutes of Health (NIH), in an effort to “characterize microbial communities found at several sites on the human body, including nasal passages, oral cavities, skin, gastrointestinal tract, and urogenital tract, and to analyse the role these microbes play in human health and disease”. The NIH-funded Human Microbiome Project Consortium has been able to map the microbial signature of normal human individuals, providing a framework for current and future studies, thus leaving open future upgrades on various disease-microbiome correlations through recent research and aiming at a deeper understanding of the disease pathophysiology [50]. Recent NGS-based studies have highlighted the gut microbiome impact on different physiologies including disease, of which the gut microbiome expressed aberrant composition as compared with that of normal individuals [51].

Although the microbiota is constantly exposed to a changing environment, its composition and function in an individual remain stable, despite disturbances. Under normal conditions, the gut microbiota represents a dynamic and symbiotic ecosystem, in a continuous relationship with the host metabolism, providing trophic and protective functions. It was revealed that alterations of the commensal flora have been involved in the pathogenesis of various illnesses, including chronic inflammation and CKD.

The CKD specific uremic milieu, due to influx of urea and other retained toxins, seems to impair the intestinal barrier function and promotes inflammation throughout the gastrointestinal tract, thus being crucial in shaping the gut microbiota in terms of structure, composition, and function. Microbial diversity is significantly damaged in CKD patients, with a decreased number of beneficial bacteria that generate short-chain fatty acids (SCFAs), a fundamental nutrient for the colonic epithelium, and an increase in bacteria that produce uremic toxins such as indoxyl sulfate, p-cresyl sulfate, and trimethylamine-N-oxide (TMAO) [52]. Uremic toxicity has also been studied by the European Toxin work group (EUTox), offering novel insights into uremic milieu by developing a classification of uremic circulating components, based on their features that affect their elimination under dialysis. Thus, among small water-soluble molecules (e.g., urea and creatinine) and peptides/proteins (e.g., *β*2-microglobulin), a group of so-called protein bound uremic retention solutes has been identified, intriguingly generated by protein fermentation in the large intestine—namely, p-cresyl sulfate and indoxyl sulfate [53].

**Figure 1:**
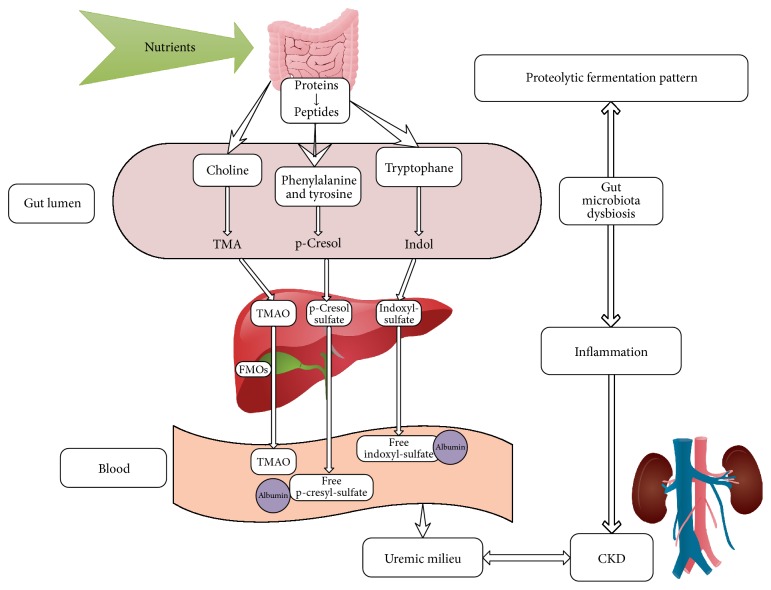
The pathway followed by the uremic metabolites (TMAO, p-cresyl sulfate, and indoxyl sulfate) in the setting of the uremic milieu, characteristic to CKD. The dysbiosis of gut microbiota contributes to the establishment of a proteolytic fermentation pattern, by enhancing the bacteria types that produce uremic toxins.

These uremic toxins were also evaluated in relation to kidney function (eGFR), and the results showed that their overexpression was correlated with an impaired renal function and an increased potential of all-cause mortality in CKD end-stage patients [54]. In addition, a direct connection was prominently revealed between increased levels of p-cresyl sulfate and poor prognosis on patients at CKD end stages; associations between indoxyl sulfate and unfavourable prognosis have been shown, as well, since it was demonstrated they share common ground, being both originated from bacterial protein fermentation in the large intestine. It was revealed that the circulating forms of these molecules are bound to albumin, competing for the same albumin-binding sites. Further studies have been conducted and have launched the theory by which the adsorption of indoxyl sulfate and p-cresyl sulfate at the intestinal level will lead to a delay in CKD progression. In light of these findings, it was optimistically hypothesized that these two molecules could be considered promising candidate biomarkers for evaluating the CKD progression [53] (see Figure 1).

It should be emphasized that renal phenotype is much broader than function impairment of kidneys, and most of the end-stage CKD patients are under multidrug therapy and dietary restrictions. Therefore, testing the associations between renal function and microbiome composition could offer accurate results when assaying on experimental models. In addition, dietary restrictions in CKD end-stage patients may be associated with limited intake of potassium, sodium, phosphate, and animal proteins, as well, also restrictions in fermentable carbohydrates. As a result, the colonic transit time is prolonged, and CKD patients undergoing dialysis are suffering though of constipation. As a consequence of diet restrictions and prolonged colonic transit, the microbiota activity moves towards a proteolytic fermentation pattern. This metabolic shift represents the explanation of significant prevalence in bacterial types processing urease, uricase, and indole and p-cresol forming enzymes [55]. Microbial diversity is significantly damaged in CKD patients, with a decreased number of beneficial bacteria that generate SCFAs and an increase in bacteria that produce uremic toxins (indoxyl sulfate, p-cresyl sulfate, and TMAO) [52].

Recent evidence suggests that several circulating metabolites released by microbiota metabolism could be linked to systemic immunoinflammatory response and kidney impairment. Thus, some metabolites generated by dietary fiber fermentation in the intestinal tract (including SCFAs) could play important roles in modulating immunity, blood pressure, and lipid metabolism. Though controversial, the SCFAs could be regarded as potential therapeutic targets and seem to represent the link between the kidney malfunction and inflammatory response [56].

Inside CKD population, the interactions work bidirectionally: on one hand, the uremic milieu has a negative impact on microbiota, altering the composition and metabolism, and on the other hand, the microbiota dysbiosis releases potential uremic toxins that are normally excreted by the kidneys; thus, both conditions further lead to a toxin avalanche exposure. The generated state is also caused by the disruption of the epithelial barrier, leading to an amplified intestinal permeability, often referred to as “leaky gut,” a condition that promotes inflammation and is encountered in CKD [57].

Intestinal inflammation and gut dysbiosis are nowadays considered as significant contributors in the setting of chronic inflammation and other CKD complications, thus explaining the gut-therapeutic novel approaches when designing CKD interventions [58].

### 4.1. Dietary Patterns in Preventing CKD Progression

Preventing the gut dysbiosis and maintaining the gut microbiota homeostasis are considered the key mechanisms for hampering the setting of chronic inflammation and CKD progression. Based on the principle that a balanced healthy microbiota is primarily saccharolytic and nutrition has significant effects on its composition, the innovative therapeutic avenues comprise special diets that successfully shape microbiota composition through a nonpharmacological approach. The Mediterranean diet, consisting mainly of carbohydrates, basically unrefined grains, fruits and vegetables, nuts, olive oil, fish, moderate red wine, dairy products, and red meat, represents one of the most promising nutritional strategies, having protective effects on CKD conditions, potentially restoring microbiota balance and slowing down CKD progression, as many studies have depicted [59–61]. Additional benefits in reducing the burden of uremic toxins, generated both by microbiota and CKD condition, were noticed under a vegetarian diet; however, increasing attention must be paid in regard to serum potassium levels [62, 63]. Other promising diets have been proposed as potential beneficent therapies, including vegan diet, DASH diet, and the modern dietary pattern, all exhibiting protective effects on both CKD progression [64, 65] or on intestinal microbiota homeostasis [62]. In contrast, the Western diet, excessively rich in proteins and low in fruits and vegetables, grains, and fibers, exerts a detrimental effect on CKD, by increasing the risk of rapid eGFR decline [66]. Along with the Western diet, other essential diets have been assessed in relation to their kidney function decline, comprising the Southern diet, DGA diet, and dal diet [67–69].

### 4.2. Prebiotics, Probiotics, and Synbiotics—Promising Therapies in Modulating Gut Microbiota in CKD

A promising therapeutic approach in combating CKD progression relies on targeting microbiota balance, by administrating prebiotics and probiotics and the mixture of both preparations into synbiotic compounds.

*Probiotics* are microorganisms that are claimed to provide beneficial effects and are defined as “live microorganisms that when administrated in adequate amounts confer a health benefit on the host” [70]. Administration of probiotics, mainly represented by Bifidobacteria, Lactobacillus, and Streptococci species, could attenuate the CKD progression. Recent studies, based on a rat model of CKD, suggest that probiotic therapy has a substantial potential in ameliorating the disease course [71]. A significant decrease in urea nitrogen circulating levels and a favourable CKD prognostic rate were reported in a multinational trial on CKD stage 3 and 4 undertaking proprietary formulation of *S. thermophilus*, L. acidophilus, and B. longum, over a period of six months. However, if these effects are due to alteration of the gut tight junction barrier remains questionable, further studies being necessary to unravel the precise mechanisms [72].

*Prebiotics* are typically specialized nondigestible plant fiber compounds that circulate undigested through the upper part of the gastrointestinal tract and enhance the activity of beneficial bacteria in the gut, presenting also a beneficial effect on CKD prognosis [73]. Prebiotics are commonly known as a type of fiber referred to as “oligosaccharides,” and the promising therapeutic candidates are represented by inulin, fructooligosaccharides, galactooligosaccharides, soyaoligosaccharides, xyloolygosacchrides, and pyrodextrins and seem to enhance the metabolic activity of microbial species like Bifidobacteria and Lactobacillus [74]. Other relevant studies focused on the effects exerted by administration of prebiotics, probiotics, and the dual approach of combining those two preparations (synbiotics) in CKD, in both patients and animal models, have been depicted in Table 1.

*Synbiotics* have been the subject of different research studies, with the term pertaining to combinations in which probiotics and prebiotics strengthen each other's activity, resulting in a synergistic effect. Recent studies highlighted that administration of synbiotics has generated favourable effects, by decreasing the circulating levels of uremic toxins, along with a restoration in microbiota balance [75]. A meta-analysis of 12 studies on the effectiveness of pre-, pro-, and synbiotics on CKD populations has reported significantly decreased levels of the two protein-bound uremic toxins (p-cresyl sulfate and indoxyl sulfate), concluding that “there is a limited but supportive evidence for the effectiveness of pre- and probiotics on reducing p-cresyl sulfate and indoxyl sulfate in the chronic kidney disease population,” but that “further studies are needed to provide more definitive findings before routine clinical use can be recommended” [76].

In conclusion, these novel promising therapeutic approaches, in which diet represents the essential factor in alleviating the disease progression, are not quite new if we go back in ancient Greece, nearly 2500 years ago, when Hippocrates postulated that “All disease begins in the gut.”

## 5. CKD and Malignancy—Dangerous Scenarios in the Framework of Inflammation

The role of inflammation in the development of cancer has been the subject of intense research over the years, since it was noted that an inflammatory milieu arises as one of the hallmark features describing the malignancy condition. There has been over 150 years since Virchow first hypothesized the relationship between the inflammatory status and carcinogenesis, based on the assumptions that cancer regularly occurs in the setting of inflammation, and additionally, that tumour biopsy specimens reveals the presence of inflammatory cells, as well. In an attempt to establish the signature of cancer, a repertoire of six hallmarks has been initially described, in which inflammation fostered multiple hallmark functions [86]. Following these established hallmarks, Fouad and Aanei proposed a more accurate definition of cancer hallmarks as “acquired evolutionary advantageous characteristics that complementarily promote transformation of phenotypically normal cells into malignant ones, and promote progression of malignant cells while sacrificing/exploiting host tissue” [87].

Nowadays, a plethora of research studies has confirmed that mitogenesis arises within an inflammatory microenvironment [88], while chronic, low-grade inflammation accompanies the disease course. The inflammatory milieu allows tumour cells to elude host immunosurveillance, resulting in subsequent angiogenesis, tumour growth, invasion, and metastasis [23, 89].

It is widely accepted that inflammation and carcinogenesis rely on similar mechanisms in terms of development, including severe cell proliferation and angiogenesis [90]. It was hypothesized that the longer the inflammation persists, the higher the possibility of genomic instability and mutations that lead to cancer. The sustained presence of inflammatory cells in the tumour milieu can stimulate tumour growth, hindering apoptosis of atypically transformed cells [91]. Peeking behind the curtain, two compliant pathways (intrinsic and extrinsic) seem to engage inflammation in cancer development. Key players of the intrinsic pathway reside in genetic modifications such as oncogene activation and tumour suppressor gene inactivation.

The principal mechanisms involved in renal carcinoma pathogenesis seem to be mediated via PI3K-AKT-mTOR, Ras-RAF-ERK, and VEGF signaling pathways, and the level of expression of the genes that are components of these pathways was positively correlated with overall survival in these patients. Therefore, further research targeting the genes and their encoded products, within these pathways, is needed to provide more insight into the involved pathways [92, 93].

The extrinsic pathway driven by inflammatory conditions generally arises and increases the risk of cancer at certain anatomical sites. Intrinsic and extrinsic factors may cooperate towards a malignant phenotype [94].

Key orchestrators of both intrinsic and extrinsic pathways consist of transcription factors (including NF-κB) that serve as a pivotal mediator of inflammatory responses (avalanche of cytokines, chemokines), being also an active player in cancer initiation, development, metastasis, and resistance to treatment [95, 96].

The inflammatory infiltrate is one of the examples underlying the inflammatory microenvironment generated by the avalanche of inflammatory mediators expressed along with the activation of this pathway.

Remarkably, NF-κB is constitutively active in both tumoural cells and tumour microenvironment and uncommonly activated via genetic alterations [97]. However, it was revealed that, in all malignancies, NF-κB acts in a cell type-specific fashion: stimulating survival genes within tumour cells and inflammation-promoting genes in components of the tumour milieu [98]. Hence, the active NF-κB molecule in cancer is acting like a double-edged sword: on one hand, mediating the immune responses by eliminating tumour cells and, on the other hand, being constitutively active in renal cancer, arising from a chronic low-grade inflammatory milieu or rarely being activated by oncogenic aberrations [99].

The CXCL12–CXCR4 signaling pathway is emerging as a novel potential therapeutic target for renal cancer, CXCR4 being overexpressed in renal malignant cells, contributing to tumour dissemination and metastasis. Blocking this pathway results in a decreased rate of metastasis and could also be effective when CXCR4 is administered in conjunction with other anticancer treatments [100].

It is well known that renal cell carcinoma (RCC) develops as one of the most immunogenic cancers, thus being able to induce an immune response naturally. Therefore, several immunotherapeutic strategies have been experienced by modulating the immune system with cytokines, vaccines, and T-cell modulating agents, having optimistic long-term results. It was revealed that administration of interleukin-2 (IL-2) in high doses could represent the first-line treatment approach for selected patients and was correlated with resilient complete remissions in treated patients [101].

The association between CKD in its end stage in patients demanding kidney transplantation and development of kidney malignancy has become well recognized. Unfortunately, there is mounting evidence that malignancy, overall or targeting kidneys, nests in even earlier stages of CKD [102]. Due to the insidious nature of CKD progression, it becomes even more difficult to diagnose these patients in their early stages, bringing yet additional burden. Remarkably, there is emerging evidence that consider CKD and renal carcinoma as interrelated, with 26%–44% of renal cell carcinoma cases bearing concomitant moderate or higher CKD at the time of diagnosis. In addition, patients suffering from renal cancer are more predisposed to CKD than the general population. Potentially involved mechanisms could include uremic immune inhibition or circulating toxin exposure in the background of a deficient renal function. Consequently, kidney tumour management has to consider the renal functional status in the decision of resecting the tumour or adopting a surveillance attitude. It was shown that RCC with low-grade tumours, arising in patients suffering from end-stage CKD, seems to manifest favourable outcome features compared to those diagnosed from the general population [103].

Although CKD is correlated with a high rate of progression towards end-stage renal disease and increased mortality, it was hypothesized that the etiology of renal decline could alter the CKD progression and overall survival. Therefore, data suggest that surgically induced CKD, including partial or total nephrectomy as a therapeutical option for renal tumour, present a lower rate in eGFR decline compared to CKD due to other medical causes [104].

A progressive relationship between pretreatment CKD and locally advanced RCC has been reported, possibly related to increased damage of functional renal parenchyma with tumour size or stage advancement [105]. Also, in an Australian population-based cohort analysis, Ahn et al. evaluated the predictors of new-onset CKD or moderate-severe CKD in patients surgically treated for T1 RCC and found out that the strongest associations were increasing age, decreased renal function (eGFR), and the tumour size, as well [106].

Regardless of the renal tumour size or stage migration, the survival rates are not encouraging over the last 15 years; however, a survival rate of 90% or more, depending on the tumour histology, is expected for the small tumours, when partial or total nephrectomy was performed [107].

In conclusion, a bidirectional relationship has been established for kidney disease and cancer, being intertwined in various ways. On one hand, malignancy has been recognized as a major complication in CKD end-stage patients, increasing the morbidity and mortality; on the other hand, anticancer therapies enhance the development of CKD [108]. Unfortunately, regardless of significant advances in therapy, RCC is nowadays among the 10 most prevalent malignancies, and the incidence is growing. Additionally, RCC has a poor prognosis, considering that up to 30% of patients present metastasis at the time of diagnosis and about 20% will further develop metastasis, even if they are undergoing therapy [109].

Despite the increasing body of evidence regarding the troubling connection between CKD and renal cancer, there is a lack of strong clinical trials in the efforts to decipher the underlying disease mechanisms and to offer novel insights towards early diagnostic and the best therapeutic approaches.

## 6. Novel Promising Biomarkers Useful in CKD Management

The advent of proteomic technologies allowed novel approaches for biomarker discovery in CKD, with the end goal being early diagnosis and prognosis of CKD progression. Candidate biomarkers include molecules that were linked to different pathways, among which tubulointerstitial injury, tubulointerstitial fibrosis, and inflammation [110–115].

In a large multicentral international study of hemodialysis patients, evaluation of CRP levels, in addition to standard inflammatory biomarkers (eGFR, albumin, WBC, and ferritin), seemed to improve the mortality prediction. The CRP level was positively and monotonically associated with mortality [116].

Another study evaluating the association between kidney function, albuminuria, and biomarkers of inflammation, in a large cohort of CKD patients, showed that plasma levels of IL-1*β*, IL-1RA, IL-6, TNF-*α*, hs-CRP, and fibrinogen were higher among participants with lower levels of estimated GFR (glomerular filtration rate). Moreover, the inflammation score was higher among the patients with lower estimated GFR and higher UACR (urine albumin to creatinine ratio). These results demonstrated that biomarkers of inflammation were inversely associated with measures of kidney function and positively with albuminuria [117]. The erythrocyte sedimentation rate, a nonspecific determinant of inflammation, has been shown to be predictive of end-stage renal disease in adolescents [118]. The level of proinflammatory cytokine IL-2 was elevated in hemodialysis patients with uremic pruritus (a common tormenting symptom among these patients) when compared to hemodialysis patient controls without pruritus [119]. The results obtained from several studies suggest that TWEAK (Tumour necrosis factor-like weak inducer of apoptosis) plays an important role in kidney injury associated with inflammation and promotes acute and chronic kidney diseases [120]. There are several studies testing different nanoconjugates that could prevent TWEAK-induced cell death and inflammatory signaling in different cell types, including renal tubular cells [121]. The results obtained from a study investigating hemodialysis patients showed that the group of patients with a specific pattern of high proinflammatory cytokines (IL-1, IL-6, and TNF-*α*) had increased mortality when compared to patients with a pattern of high T-cell regulatory or anti-inflammatory parameters (IL-2, IL-4, IL-5, IL-12, CH50, and T-cell number) [122]. Leptin is an adipose tissue-derived hormone shown to be associated to several inflammatory factors related to CKD. In vivo studies demonstrated that infusion of recombinant leptin into normal rats for 3 weeks results in the development of glomerulosclerosis. Moreover, higher plasma leptin levels are associated with CKD, and the authors of these studies sustain that leptin may explain part of the reported association between obesity and kidney disease [123].

Kidney injury molecule-1 (KIM-1), a type 1 transmembrane protein, has been shown to be upregulated in dedifferentiated proximal tubule epithelial cells upon ischemic or toxic injury but is undetectable in healthy kidneys or urine [124–127]. Urinary KIM-1 has been shown to predict renal injury before changes in eGFR were detectable [128, 129].

Neutrophil gelatinase-associated lipocalin (NGAL) is a protein expressed by tubular epithelial cells and neutrophils, and its expression levels were shown to predict disease severity [130, 131]. However, NGAL did not significantly improve risk prediction of progression outcomes compared to known CKD progression risk factors [132].

Epidermal growth factor (EGF) plays a role in tubular cell repair after tubulointerstitial injury. Urinary EGF expression was found to be correlated with GFR [133], and it improves CKD progression prediction when added to a conventional model including eGFR and albuminuria [134].

A candidate marker of renal fibrosis is matrix metalloproteinase-9 (MMP-9), which was found to be elevated in the urine and plasma of CKD patients compared to controls [135, 136]. Additionally, circulating MMP-9 levels improved CKD progression predictability when added to a model of conventional risk factors and eGFR [137].

Chronic low-grade inflammation is proposed to play an important role in the initiation and progression of CKD, and several candidate biomarkers have been suggested to predict GFR, as well as contribute directly to CKD progression [114, 115]. Soluble urokinase-type plasminogen activator receptor (suPAR) is involved in the pathogenesis of kidney disease. A low suPAR concentration was shown to be associated with the remission of CKD and the reduction of proteinuria (23138488). Furthermore, higher plasma suPAR was connected with CKD progression, as indicated by a stronger decline in eGFR [115]. Other inflammatory markers associated with CKD include tumour necrosis factor alpha receptor-1 and -2 (TNFR1 and TNFR2) and monocyte chemoattractant protein-1 (MCP-1). TNFR1 was found to be a strong prediction of CKD progression to ESRD [114], while circulating TNFR1 and TNFR2 were found to predict stage 3 CKD in type 1 diabetes patients. Urinary MCP-1 levels were elevated for CKD patients compared to controls [138] and were found to correlate with the rate of GFR decline [139].

Another study analysing the levels of MCP-1, MCSF, and neopterin in the serum and urine of children with CKD showed that MCP-1 levels are increased in early stages of this disease, suggesting that the inflammatory process precedes the tubular dysfunction [140].

In view of the increasing number of novel potential candidate biomarkers, advanced high-throughput research platforms are needed in order to refine the CKD diagnosis, monitoring, and follow-up.

## 7. Advances in Proteomic Approaches in Searching for an Ideal Biomarker

Although substantial improvements have been made in clinical care, CKD remains a major public health burden, affecting 10–15% of the population, and its prevalence is constantly growing [141]. Regardless of its etiology, CKD is defined as a “silent epidemic” disease and persistent, with low-grade inflammation reflecting a common feature in these patients. Due to its insidious nature, CKD is rarely diagnosed in early stages, as clinical symptoms occur only when kidney function has been irreversibly damaged (decreased eGFR). Unfortunately, current clinical approaches have become useful only in diagnosis of advanced CKD stages. Simply stated, once developed, CKD persists throughout the rest of the patient's life, and the single most feasible solution is likely linked to an early intervention, before irreversible nephron damage occurs [142]. In addition, nephrology lags behind other medical disciplines in terms of number, size, and quality of clinical trials undertaken, thus emerging provocative global action plans in order to improve the management of CKD and design novel therapeutic approaches to alleviate or even halt the progression of the disease [141].

In this scenario, a huge step forward was made by the increasing progression of omics approaches, designed for identification of biomarkers useful for early diagnostic and follow-up, thus exploring their potential for clinical implementation [143, 144]. In the era of omics, proteomics has risen, providing novel insights into disease mechanisms and therefore holds the promise of improving the life quality of CKD patients.

Advancements in the field of proteomics were possible by adopting a vast array of state-of-the-art technologies. Initially, two-dimensional (2D) gel electrophoresis was used, rapidly being improved by the development of two-dimensional differential gel electrophoresis (2D-DIGE), completed afterwards by employing liquid chromatography (LC) coupled with mass spectrometry (MS), enabling though untargeted protein identification. During recent years, capillary-electrophoresis (CE)-MS has been developed, combining both CE and MS advantages, providing high separation efficiency and molecular mass information within one single assay. Implementing the matrix-assisted laser desorption/ionization (MALDI) platform, by using laser energy absorbing matrix, is capable of generating ions from large molecules with minimal fragmentation, thereby moving the boundaries above. Proteomics aims to characterize the huge information flow mediated by proteins within the cell, by analysing the signaling pathways, interactions, and networks, thus enabling identification of disease specific biomarkers in order to illustrate a detailed proteomic signature for a better understanding of the molecular interactions underlying the pathogenesis of the disease. Assessing various biomarkers on multiplex proteomic platforms (Luminex xMAP array, microarrays, etc.) could unravel novel insights in deciphering the disease-specific molecular mechanisms, offering panels of biomarkers for improving the diagnosis and therapy towards a personalized approach [143, 145–147]. In the context of CKD and renal diseases, various proteomic studies have been designed, and the results were promising. Recent findings performed on MALDI suggested that molecular signatures could be generated, being capable of distinguishing between kidney disease and normal controls [148]. Siwy et al. analysed several potential urinary peptides to differentiate between distinct types of CKD, generated by capillary electrophoresis coupled to mass spectrometry [149]. Such findings are corroborated with other study results and confirm the utility of some of these urinary peptides as specific biomarkers [150]. Good et al. have developed a CKD classifier (CKD273), comprising 273 urinary peptides, specially designed for a better stratification in these patients [151]. CKD273 represents a multidimensional urinary biomarker which helps predict the renal function impairment [152]. Other studies aimed at predicting the risk of CKD progression, by determining patterns of protein expressions using mass spectrometry approaches (SELDI-TOF) [153]. CKD273 has recently received a letter of support from the US Food and Drug Administration (FDA), being now implemented in the CKD management [154]. Furthermore, CKD databases have been created; thus, KUPNetViz represents an interactive and flexible biological network tool for multiomics datasets, in the field of kidney diseases, providing biological network snapshots of the complex integrated data of the KUPKB (Kidney and Urinary Pathway Knowledge Base), thus creating the premises of generating novel in silico theories [155]. Furthermore, a CKD database (CKDdb) has been developed due to the vast amount of data generated by using high-throughput omics technologies. CKDdb represents an integrated and clustered information resource; featuring data from CKD published studies will result in deeper understanding of the molecular mechanism modulating CKD progression [156].

The translation of omics findings to clinical settings is challenging, since an ideal biomarker has not been discovered yet, thus being recommended to adopt a two-stage approach: firstly, the identification step, followed by the validation, applicable only in the framework of a well-defined clinical question and a specific phenotype [157].

## 8. Conclusions

Despite being a “silent epidemic” disease, CKD is now recognized as one of the major public health burden, affecting 10–15% of the population, and its prevalence is constantly growing. Mounting evidence suggests implication of inflammation in CKD pathophysiology, thereby shifting the perception of inflammation as no longer a new risk factor but rather a traditional one linked to morbidity and mortality in these patients. The pathophysiology of inflammation may not be the same in CKD patients; nevertheless, a persistent, low-grade inflammation has been established as a hallmark feature of CKD.

Among various factors that contribute to the setting of an inflammatory milieu in the context of CKD, the inflammasome has recently become the focus of extensive research, gaining recognition for its key role in the pathogenesis of CKD and its complications. As such, the inflammasome represents an attractive potential therapeutic target in renal diseases. Another underestimated source of smouldering inflammation related to CKD was assigned to gut microbiota dysbiosis, a condition intensively studied, since it was postulated that may represent the starting point of many diseases, including malignancy. Modulating the microbiota balance has become a subject of intense research; therefore, different dietary patterns have been proposed, along with administration of pre-, pro-, and synbiotics, with quite remarkable results.

In this scenario, a huge step forward was made by the increasing progression of omics approaches, specially designed for identification of biomarkers useful for early diagnostic and follow-up. Advances in proteomics, in searching for the ideal biomarker, have become increasingly popular over the last decades, offering novel insights in deciphering the CKD mechanisms, thus moving the boundaries forward. The identification of novel biomarkers using high-throughput technologies will provide the molecular signature of the disease, with impact on early diagnosis, monitoring, and prognosis.

Understanding the role of inflammation in the setting of CKD will foster the development of therapeutic strategies in order to treat and even prevent the underlying inflammation, thus improving CKD outcomes.

## Figures and Tables

**Table 1:** Effects of administrating pro-, pre-, or synbiotics in CKD.

Novel therapeutic targets	Effects on CKD	Reference
Probiotics—Lactobacillus acidophilus	Nitrosodimethylamine levels decreased, and serum dimethylamine levels dropped (on humans).	[77]
Probiotics—*Bacillus pasteurii* or *Lactobacillus sporogenes*	Enhanced survival in nephrectomized rats while slowing the progress of renal injury (rat model).	[78]
Probiotics—*Sprosarcina pasteurii*	Reduced blood urea-nitrogen levels and significantly prolonged the lifespan of uremic animals (rat model).	[79]
Probiotics—oral sorbent charcoal AST-120	Delay in the progression of CKD but also in cardiovascular diseases (rat model).	[80]
Probiotics—*Bifidobacterium longum*	Reduced serum levels of indoxyl sulfate by correcting the intestinal microflora (on humans).	[81]
Probiotics—*Bifidobacterium longum*	Decreased serum levels of homocysteine, indoxyl sulfate, and triglyceride (on humans).	[82]
Prebiotics—oligofructose-enriched inulin	Significantly reduced p-cresyl sulfate generation rates (on humans).	[83]
Prebiotics—resistant starch	Reduced plasma levels of indoxyl sulfate and p-cresol sulfate (on humans).	[84]
Synbiotics	Decreased serum p-cresol sulfate and the stool microbiome modified (on humans).	[75]
Synbiotics	Normalization of bowel habits and a decrease of serum p-cresol levels (on humans).	[85]
